# Regulatory Fibroblast‐Like Synoviocytes Cell Membrane Coated Nanoparticles: A Novel Targeted Therapy for Rheumatoid Arthritis

**DOI:** 10.1002/advs.202204998

**Published:** 2022-12-12

**Authors:** Yuan Liu, Peishi Rao, Hongyan Qian, Yesi Shi, Shiju Chen, Jingying Lan, Dan Mu, Rongjuan Chen, Xinwei Zhang, Chaoqiong Deng, Gang Liu, Guixiu Shi

**Affiliations:** ^1^ Department of Rheumatology and Clinical Immunology The First Affiliated Hospital of Xiamen University Xiamen 361001 China; ^2^ School of Medicine Xiamen University Xiamen 361103 China; ^3^ Xiamen Municipal Clinical Research Center for Immune Disease Xiamen 361001 China; ^4^ State Key Laboratory of Molecular Vaccinology and Molecular Diagnostics Center for Molecular Imaging and Translational Medicine School of Public Health Xiamen 361001 China; ^5^ Department of Rheumatology and Immunology Peking University People's Hospital Beijing 100044 China

**Keywords:** cell membrane coated nanoparticle, regulatory fibroblast, rheumatoid arthritis

## Abstract

Fibroblast‐like synoviocytes (FLS) are the main cell component in the inflamed joints of patients with rheumatoid arthritis (RA). FLS intimately interact with infiltrating T cells. Fibroblasts have potent inhibitory effects on T cells, leading to the resolution of inflammation and immune tolerance. However, this “regulatory” phenotype is defect in RA, and FLS in RA instead act as “proinflammatory” phenotype mediating inflammation perpetuation. Signals that orchestrate fibroblast heterogeneity remain unclear. Here, it is demonstrated that different cytokines can induce distinct phenotypes of FLS. Interferon‐gamma (IFN‐*γ*) is pivotal in inducing the regulatory phenotype of FLS (which is termed FLS^reg^) characterized by high expressions of several inhibitory molecules. Rapamycin enhances the effect of IFN‐*γ* on FLS. Based on the characteristics of FLS^reg^, a novel biomimetic therapeutic strategy for RA is designed by coating cell membrane derived from FLS^reg^ induced by IFN‐*γ* and rapamycin on nanoparticles, which is called FIRN. FIRN show good efficacy, stability, and inflammatory joint targeting ability in an RA mouse model. The findings clarify how fibroblast phenotypes are modulated in the inflammatory microenvironment and provide insights into novel therapeutic designs for autoimmune diseases based on regulatory fibroblasts.

## Graphical abstract

This study demonstrates an in vitro induction method for regulatory fibroblast‐like synoviocytes (FLS) phenotype (FLS^reg^) by interferon‐gamma and rapamycin. Based on the characteristics of FLS^reg^, a novel biomimetic therapeutic strategy for rheumatoid arthritis (RA) is proposed by coating cell membrane derived from FLS^reg^ on nanoparticles, which shows good efficacy, stability, and inflammatory joint targeting ability in the RA mouse model.

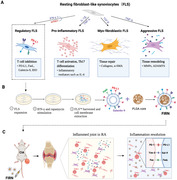

## 1 Introduction

Rheumatoid arthritis (RA) is one of the most common systemic autoimmune diseases, affecting ≈0.5 to 1% of the population worldwide.^[^ ^1^ ^]^ RA is characterized by persistent synovial inflammation in multiple joints, leading to joint damage and involvement of vital organs, such as the heart, lungs, and kidneys.^[^ ^2^ ^]^ Although considerable advances in anti‐cytokines therapy and Janus kinase (JAK) inhibitors have improved outcomes, off‐joint toxicity remains a considerable challenge for these traditional drugs, given the prevalent expression of these drug targets. Despite treatment according to the current management recommendations, a significant proportion of patients with RA remains symptomatic and is considered as “difficult‐to‐treat RA.”^[^ ^3^ ^]^ Exploration of new RA therapeutic strategies is important.

Persistent inflammation, which does not resolve, is the mainstay of RA pathogenesis. Persistently activated T cells play a pivotal role.^[^ ^4^ ^]^ High‐throughput unbiased technologies, such as mass cytometry and single cell RNA sequencing (scRNA‐seq), have revealed that among T cells, the programmed cell death protein 1 (PD‐1) positive T cells, including the CXCR5^+^ CD4^+^ T cells (follicular helper T cells, TFH) and CXCR5^−^ CD4^+^ T cells (peripheral T helper cells, TPH), are highly expanded in the inflamed synovium of patients with RA.^[^ ^5^ ^]^ Under normal physiological conditions, immune responses are monitored by immune checkpoints to prevent autoimmunity. However, despite the high expression of PD‐1, T cells in RA are not exhausted and are actively involved in its pathogenesis.^[^ ^5b^ ^]^ The PD‐1/PD‐ligand 1 (PD‐L1) axis is essential in maintaining immune homeostasis by limiting activated T cells.^[^ ^6^ ^]^ However, why PD‐1^+^ T cells in RA remain persistently activated instead of receiving sufficient inhibitory signal from PD‐L1 to limit their function is unclear.

Fibroblast‐like synoviocytes (FLS) are the main cell component in the inflamed synovium. FLS intimately interact with T cells in RA.^[^ ^7^ ^]^ FLS are the most important effector cells in RA pathogenesis; these cells increase in number and become a prominent component of the destructive pannus that characterizes synovium in RA. In addition to acting as passive effector cells, recent studies have revealed the role of FLS in sustaining inflammation in RA by promoting T cell activation and Th17 differentiation, making FLS an attractive new therapeutic target in RA.^[^ ^8^ ^]^ FLS in RA act as a “proinflammatory” and “aggressive” phenotype characterized by the expression of several disease‐relevant cytokines, chemokines, and extracellular matrix remodeling factors, which leads to inflammation and destruction of joints.^[^ ^9^ ^]^ However, fibroblasts in other microenvironments presented as a “regulatory” phenotype with potent inhibitory effects on activated T cells by high expression of inhibitory molecules such as PD‐L1.^[^ ^10^ ^]^ This phenotype appears to be defect in the RA microenvironment, which may contribute to the persistent activation of T cells. Factors driving the differentiation of “regulatory” phenotype of FLS are still unclear. Understanding the signals orchestrating FLS heterogeneity may provide insights into the development of therapeutic strategies targeting expanded PD‐1^+^ T cells in RA.

Here, we demonstrated that the regulatory phenotype of FLS, characterized by high expression of PD‐L1 and several other inhibitory molecules, such as Galectin‐9 (Gal‐9) and Fas Ligand (FasL), can be induced in vitro by interferon‐gamma (IFN‐*γ*) plus rapamycin. To avoid the proinflammatory effect of IFN‐*γ*, we proposed a novel biomimetic therapeutic strategy for RA by coating nanoparticles with cell membrane derived from regulatory FLS induced by IFN‐*γ* plus rapamycin, which we called FIRN. FIRN demonstrated good efficacy, stability, and inflammatory joint targeting ability in an RA mouse model. The findings provided insights into novel therapeutic designs for autoimmune diseases based on regulatory fibroblasts.

## 2 Results

### 2.1 PD‐1^+^CD4^+^ T Cells Are Expanded in the Inflamed Joint of Patients with RA

**Figure 1:**
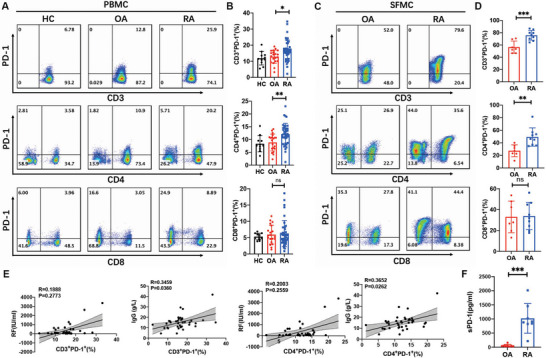
PD‐1^+^CD4^+^ T cells are expanded in inflamed joint of patients with RA. A,B) Peripheral blood mononuclear cells (PBMCs) from patients with rheumatoid arthritis (RA, *n* = 40), osteoarthritis (OA, *n* = 20), and health controls (HC, *n* = 11) were analyzed by fluorescence‐activated cell sorting (FACS) to determine the percentage of PD‐1^+^ T cells among total T cells. C,D) Synovial fluid mononuclear cells (SFMC) from patients with RA (*n* = 9) and OA (*n* = 7) were analyzed by FACS to determine the percentage of PD‐1^+^ T cells among total T cells. E) Correlations of clinical parameters with PD‐1^+^CD3^+^ T cells percentage and PD‐1^+^CD4^+^ T cells percentage in PBMCs from patients with RA. F) Soluble PD‐1 in synovial fluid of patients with RA (*n* = 8) and OA (*n* = 8) was detected by ELISA. The data are presented as the mean ± s.e.m. Two‐sided unpaired *t*‐test and Spearman correlation analysis were used to calculate *P*‐value (* *P* < 0.05, ** *P* < 0.01, *** *P* < 0.001).

Among T cells, the PD‐1^+^CD4^+^ T cells, including the CXCR5^+^ TFH and CXCR5^−^ TPH cells, are highly expanded in the inflamed synovium of patients with RA and are intimately involved in the pathogenesis of RA.^[^ ^5a,b^ ^]^ We first investigated the presence of PD‐1^+^ T cells in patients with RA. Compared to patients with osteoarthritis (OA) and healthy controls (HC), the percentage of PD‐1^+^CD4^+^ T cells in the total T cell population increased significantly in peripheral blood mononuclear cells (PBMC) from patients with RA (**Figure** **1**A,B). Additionally, PD‐1^+^CD4^+^ T cell prevalence was positively correlated with serum immunoglobulin G (IgG) levels (Figure 1E). More than half of the T cells were PD‐1^+^ in synovial fluid from inflamed joints in patients with RA. Additionally, the percentage of PD‐1^+^CD4^+^ T cells among the total T cells was also significantly higher in RA patients compared with OA patients (Figure 1C,D). We also investigated the level of soluble PD‐1 (sPD‐1) in synovial fluid from inflamed joints of patients with RA. Levels of sPD‐1 in synovial fluid were significantly higher in patients with RA than in patients with OA. Therefore, sPD‐1 may interfere with the interaction of PD‐1^+^CD4^+^ T cells with PD‐L1, thereby leading to relative PD‐L1 insufficiency in limiting the expansion of PD‐1^+^CD4^+^ T cells.

### 2.2 IFN‐*γ* Can Induce Regulatory Properties of FLS (FLS^reg^) Characterized by High Expression of PD‐L1

**Figure 2:**
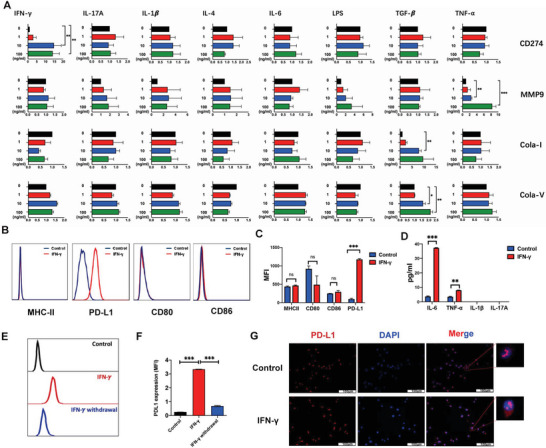
Different phenotypes of fibroblast can be induced by cytokines and IFN‐*γ* can induce regulatory phenotype of FLS. A) FLS were treated with different concentrations of TNF‐*α*, IL‐6, IL‐1*β*, IFN‐*γ*, IL‐17A, IL‐4, TGF‐*β*, and LPS for 24 h. The relative expression of CD274, MMP9, Collagen I, and Collagen V in FLS was determined by RT‐PCR. B) Expression of MHC‐II, PD‐L1, CD80, and CD86 in IFN‐*γ* (100 ng mL^−1^) treated FLS were detected by FACS. C) MFI of MHC‐II, PD‐L1, CD80, and CD86 expression. D) Inflammatory cytokines including IL‐6, TNF‐*α*, IL‐1*β*, and IL‐17A in the supernatant of FLS treated with 100 ng mL^−1^ IFN‐*γ* for 24 h were detected by ELISA. E,F) IFN‐*γ* (100 ng mL^−1^) were used to treat FLS for 24 h and then withdrawn for another 24 h. PD‐L1 was detected by FACS. G) Fluorescent images of FLS and IFN‐*γ* stimulated FLS. Red represents PD‐L1 and blue represents nuclei. (Scale bars, 100 µm; *n* = 3 independent experiments). *P*‐values were calculated using two‐sided unpaired *t*‐test (* *P* < 0.05, ** *P* < 0.01, *** *P* < 0.001).

Several studies have described the immunosuppressive/regulatory phenotype of fibroblasts characterized by the expression of multiple inhibitory molecules, such as PD‐L1, and the potent inhibitory effects on activated T cells.^[^ ^10^ ^]^ However, FLS in RA appeared to lose this phenotype and instead presented as a proinflammatory phenotype. The upstream signals driving the heterogeneity of fibroblasts phenotypes are unclear. Fibroblasts have high phenotypic plasticity,^[^ ^11^ ^]^ which may be modulated by different cytokines.^[^ ^12^ ^]^ Inflammatory joints in patients with RA are characterized by high levels of multiple cytokines. We investigated the effect on FLS of cytokines commonly presented in the inflammatory joints of patients with RA, including tumor necrosis factor‐alpha (TNF‐*α*), IFN‐*γ*, interleukin (IL)‐6, IL‐1*β*, IL‐17A, IL‐4, transforming growth factor‐beta (TGF‐*β*), and Toll‐like receptor 4 (TLR4) ligand (lipopolysaccharide, LPS). FLS demonstrated high plasticity and could differentiate into subsets with distinct functional characteristics after specific cytokine stimulation (**Figure** **2**A and Figure S1, Supporting Information). Most of these inflammatory cytokines, including IFN‐*γ*, TNF‐*α*, and IL‐17A, can induce the proinflammatory characteristics of FLS by increasing IL‐6 production (an important proinflammatory cytokine in RA pathogenesis that can induce Th17 differentiation)^[^ ^13^ ^]^ (Figure S1, Supporting Information). TGF‐*β* induced the myofibroblastic phenotype by prominently upregulating collagen expression, and TNF‐*α* induced the aggressive phenotype by upregulating expressions of several matrix metalloproteins (MMPs).

Among these cytokines, IFN‐*γ* was the only cytokine that significantly upregulated PD‐L1(CD274) expression in FLS (Figure 2A). PD‐L1 participates in the negative regulation of the T cell response; this IFN‐*γ*‐stimulated FLS (IFN‐*γ*‐FLS) appeared to present a T cell regulatory phenotype, which we termed FLS^reg^. To confirm the FLS^reg^ phenotype of IFN‐*γ*‐FLS, we further investigated the expression of antigen‐presenting and co‐stimulatory molecules in IFN‐*γ*‐FLS by fluorescence‐activated cell sorting (FACS). Expressions of major histocompatibility complex II (MHC‐II), cluster of differentiation (CD) 80, CD86, and PD‐L1 were all low on the cell surfaces of unstimulated FLS (resting FLS) (Figure 2B). PD‐L1 expression substantially increased after stimulation with IFN‐*γ* (Figure 2B,G), while no notable changes in MHC‐II, CD80, and CD86 were observed (Figure 2B,C). However, the effect of IFN‐*γ* in inducing PD‐L1 expression on FLS was temporary, as the expression level of PD‐L1 on IFN‐*γ*‐stimulated FLS quickly decreased after IFN‐*γ* withdrawal (Figure 2E,F). Meanwhile, IFN‐*γ* induced IL‐6 production by FLS (Figure 2D), demonstrating a proinflammatory aspect of IFN‐*γ*, which indicated its dual effects on FLS.

### 2.3 IFN‐*γ* Can Increase Expressions of Several Other Inhibitory Molecules on FLS in Addition to PD‐L1

**Figure 3:**
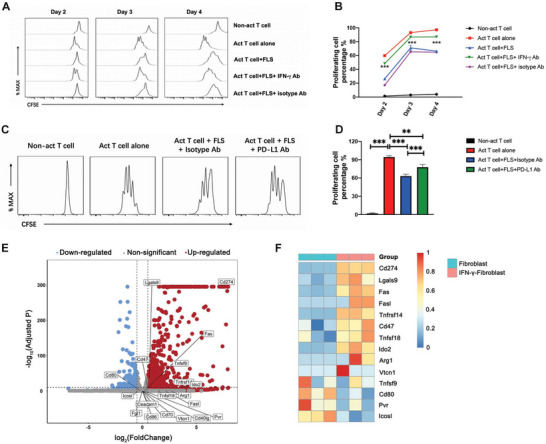
IFN‐*γ* can upregulate several inhibitory molecules on fibroblast besides PD‐L1. A,B) Proliferation rate of T cells activated by anti‐CD3/CD28 (1 µg mL^−1^) was limited when co‐cultured with FLS and the effect was blocked by IFN‐*γ* antibody (10 µg mL^−1^). C,D) Proliferation rate of T cells activated by anti‐CD3/CD28 (1 µg mL^−1^) was limited when co‐cultured with FLS and the effect was partly blocked by PD‐L1 antibody (100 µg mL^−1^). E) Volcano plot of differential gene expression in fibroblast during IFN‐*γ* stimulation. Red indicates genes with significantly increased expression during IFN‐*γ* stimulation (adjusted *P* < 0.01 and log2 expression ratio >0.5). Blue indicates genes with significantly decreased expression during IFN‐*γ* stimulation (adjusted *P* < 0.01 and log2 expression ratio <0.5). F) Expression of several T cells inhibitory and stimulatory molecules on fibroblast after IFN‐*γ* stimulation revealed by RNA‐seq is shown in the heatmap. *P*‐values were calculated using two‐sided unpaired *t*‐test (* *P* < 0.05, ** *P* < 0.01, *** *P* < 0.001). Act T cell: Activated T cell.

Several studies have reported that fibroblasts can limit activated T cell proliferation.^[^ ^14^ ^]^ We further investigated the role of IFN‐*γ* in inducing the T cell inhibitory function of FLS by blocking IFN‐*γ* during the co‐culture of FLS with activated T cells. FLS showed potent inhibitory ability to limit activated T cell proliferation. However, this ability was greatly decreased when IFN‐*γ* was blocked in the co‐culture system (**Figure** **3**A,B), confirming the vital role of IFN‐*γ* in inducing the FLS^reg^ phenotype. As IFN‐*γ* can induce PD‐L1 expression, we further investigated the role of PD‐L1 in the FLS^reg^ mediated inhibition of T cells. After blocking PD‐L1 during the co‐culture of FLS with activated T cells, the inhibitory effect of FLS on T cells was diminished (Figure 3C,D), but was not fully reversed, indicating that there might be other inhibitory molecules on FLS induced by IFN‐*γ*. We then performed mRNA sequencing on fibroblasts as well as IFN‐*γ*‐activated fibroblasts and analyzed the increased molecules related to immunological suppression (Figure 3E,F). Among the reported T cells stimulatory and inhibitory molecules, PD‐L1 was most significantly upregulated by IFN‐*γ* stimulation. In addition to PD‐L1, IFN‐*γ* stimulation can also increase several other inhibitory molecules on fibroblasts, such as Gal‐9 (Lgals9) and Fas/FasL pathway, indicating that IFN‐*γ* induced FLS^reg^ can inhibit activated T cells in a multi‐targeted manner.

### 2.4 Effect of IFN‐*γ* on FLS^reg^ Induction Can Be Further Enhanced by Rapamycin

**Figure 4:**
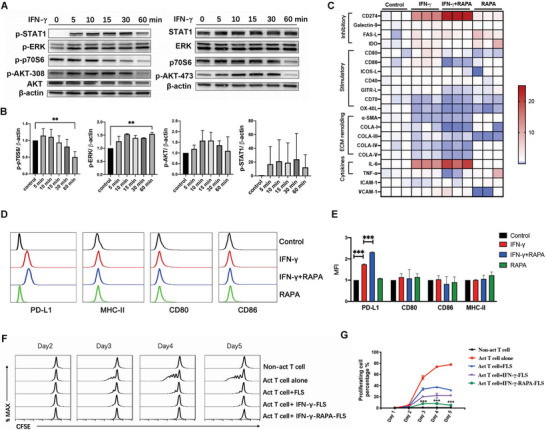
Effect of IFN‐*γ* on FLS can be enhanced by rapamycin. A,B) Cell signaling pathways activated by IFN‐*γ* (100 ng mL^−1^) in FLS were detected by western blotting. C) The relative mRNA expression determined by RT‐PCR of the co‐stimulatory molecules, extracellular matrix, and inflammatory cytokines in FLS treated with IFN‐*γ* (100 ng mL^−1^), rapamycin (10 µm), or IFN‐*γ* (100 ng mL^−1^) plus rapamycin (10 µm) after 24 h are shown in the heat map. D) Expression of MHC‐II, PD‐L1, CD80, and CD86 in IFN‐*γ* (100 ng mL^−1^), rapamycin (10 µm), or IFN‐*γ* (100 ng mL^−1^) plus rapamycin (10 µm) treated FLS were detected by FACS. E) MFI of MHC‐II, PD‐L1, CD80, and CD86 expression. F,G) IFN‐*γ* and rapamycin pre‐treated FLS showed more potent effect on inhibiting the proliferation of T cells activated by anti‐CD3/CD28 antibody (1 µg mL^−1^). The data are presented as the mean ± s.e.m. (*n* = 3 independent experiments). *P*‐values were calculated using two‐sided unpaired *t*‐test (* *P* < 0.05, ** *P* < 0.01, *** *P* < 0.001). RAPA: rapamycin; Act T cell: Activated T cell.

We then sought to dissect the intracellular signaling pathway mediating the effect of IFN‐*γ* on FLS. The activity of signaling molecules, including signal transducer and activator of transcription 1 (STAT1), protein kinase B (Akt), extracellular signal‐regulated kinase (Erk), and mammalian target of rapamycin (mTOR), in FLS stimulated with IFN‐*γ* was analyzed. Exposure to IFN‐*γ* induced phosphorylation of STAT1, Akt, and Erk, while inhibiting the activity of p70 S6 kinase (**Figure** **4**A,B), a downstream translational target used as a readout of mTOR complex 1 (mTORC1) activity. We then investigated the role of mTORC1 in fibroblast phenotype modulation by IFN‐*γ* via pharmacological inhibition of mTORC1 with rapamycin. We found that the effects of IFN‐*γ* on the expression of inhibitory molecules on FLS can be further enhanced by rapamycin, while no obvious change in these molecules was observed under rapamycin stimulation alone (Figure 4C). The expression level of PD‐L1 in stimulated FLS was further investigated by FACS (Figure 4D,E). PD‐L1 expression was significantly higher in IFN‐*γ* plus rapamycin activated FLS (IFN‐*γ*‐RAPA‐FLS) than in IFN‐*γ* activated FLS (IFN‐*γ*‐FLS), and rapamycin stimulation alone had no significant effect on PD‐L1 expression. These findings indicated that rapamycin could amplify the T cell regulatory function of FLS induced by IFN‐*γ*.

Next, we co‐cultured activated T cells with FLS, IFN‐*γ* activated FLSs (IFN‐*γ*‐FLSs), and IFN‐*γ* plus rapamycin activated FLSs (IFN‐*γ*‐RAPA‐FLSs). IFN‐*γ*‐RAPA‐FLS demonstrated the most prominent inhibitory effect on activated T cell proliferation (Figure 4F,G). These results indicated the potential use of the FLS^reg^ induced by IFN‐*γ* and rapamycin, which is characterized by high expression of PD‐L1 and several other inhibitory molecules, to target activated T cells in RA therapy.

### 2.5 FIRN Can Retain Inhibitory Effects of FLS^reg^ on T Cells and Separate Dual Effects of IFN‐*γ* on FLS

Studies have indicated that fibroblasts have high phenotypic plasticity.^[^ ^11^ ^]^ Our data showed that PD‐L1 expression on FLS decreased rapidly after IFN‐*γ* withdrawal (Figure 2E). This finding indicates that FLS^reg^ induced in vitro might lose the immunosuppressive phenotype in the inflammatory microenvironment in vivo, thus impeding the application of in vitro induced FLS^reg^ in RA treatment. Indeed, in mice with collagen‐induced arthritis (CIA), an animal model of RA, IFN‐*γ*‐FLS, and IFN‐*γ*‐RAPA‐FLS treatment did not demonstrate significant improvement in arthritis (Figure S2, Supporting Information).

**Figure 5:**
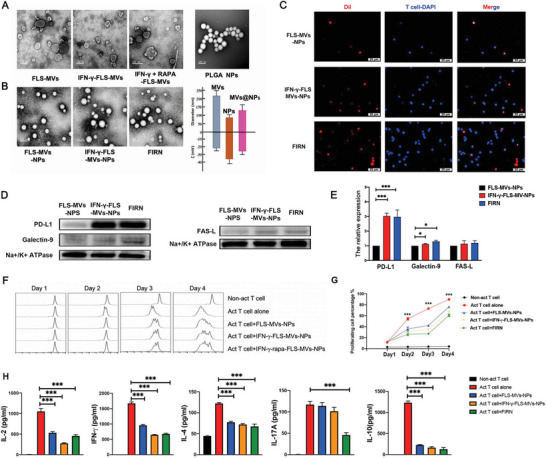
IFN‐*γ*‐rapamycin‐FLS derived cell membrane vesicles coated nanoparticles (FIRN) can bind and inhibit the proliferation and function of T cells. A) Cell membrane and membrane coated nanoparticles derived from FLS, IFN‐*γ*‐FLS, and IFN‐*γ*‐RAPA‐FLS were observed by transmission electron microscopy (TEM). B) Dynamic light scattering measurement of MVs, NPs, and MV‐NPs diameter and zeta potential (*ζ*). C) Fluorescent images of activated T cells stimulated with anti‐CD3/CD28 antibodies (1 µg mL^−1^) for 72 h after incubation with MVs‐NPs. Red represents MVs‐NPs (Dil labeled), and blue represents nuclei of T cells (Scale bars, 25 µm). D,E) PD‐L1, Galectin‐9, and FasL expression level on cell membrane vesicles derived from FLS under different stimulation coated nanoparticles (MVs‐NPs) were detected by western blotting. F,G) T cell labeled with carboxyfluorescein succinimidyl ester (CFSE) and activated by anti‐CD3/CD28 antibodies (1 µg mL^−1^) were co‐cultured with MVs‐NPs (10 µg mL^−1^). Proliferation rate of T cells was detected by FACS. H) Cytokines produced by T cells incubated with MVs‐NPs (50 µg mL^−1^) were detected by ELISA. The data are presented as the mean ± s.e.m. (*n* = 3 independent experiments). *P*‐values were calculated using two‐sided unpaired *t*‐test (* *P* < 0.05, *** *P* < 0.001). MVs‐NPs: Cell membrane vesicles coated nanoparticles; FIRN: IFN‐*γ*‐rapamycin stimulated FLS derived cell membrane vesicles coated nanoparticles; Act T cell: Activated T cell.

To facilitate the therapeutic application of FLS^reg^ induced by IFN‐*γ* and rapamycin in vitro, we sought to find a way to retain the immunosuppressive effect of FLS^reg^ steady and avoid the proinflammatory effect of IFN‐*γ* in IL‐6 induction. While the immunosuppressive effect of IFN‐*γ* on FLS appeared to be mainly translated on the cell membrane by upregulating several inhibitory molecules on the cell membrane of FLS, we hypothesized that the use of cell membranes derived from FLS stimulate with IFN‐*γ* plus rapamycin might be an effective treatment strategy be designed for RA based on FLS^reg^. This strategy may retain the anti‐inflammatory effect of IFN‐*γ* while eliminating the proinflammatory effect of IFN‐*γ* and sustaining the phenotype of FLS^reg^ induced in vitro. We then generated cell membrane nanovesicles (MVs) derived from FLS stimulated with IFN‐*γ* plus rapamycin (**Figure** **5**A). To investigate the immunoregulatory effect of MVs derived from FLS^reg^, we incubated IFN‐*γ*‐RAPA‐FLS derived MVs with T cells activated by anti‐CD3 and anti‐CD28 in vitro. IFN‐*γ*‐RAPA‐FLS derived MVs showed potent inhibitory effects on T cell proliferation and cytokine production (Figure S3, Supporting Information).

Cell membrane coated nanoparticles have been an exciting development in biomimetic nanoengineering in recent years, facilitating the development of targeted therapeutics for biologically complex applications involving multifactorial interfacing mechanisms that have not yet been fully elucidated.^[^ ^15^ ^]^ We then generated IFN‐γ‐RAPA‐FLS cell membrane coated nanoparticles, which we termed FIRN, by coating cell membrane nanovesicles derived from FLS stimulated by IFN‐*γ* plus rapamycin onto poly (lactic*‐co‐*glycolic acid) (PLGA) polymeric cores. Transmission electron microscopy (TEM) images of FIRN following uranyl acetate staining typically displayed a spherical core–shell structure, demonstrating that the membrane was successfully enveloped around the PLGA cores (Figure 5B). Dynamic light scattering (DLS) measurements revealed that the hydrodynamic diameter of the FIRN increased compared to that of the uncoated PLGA cores, and the surface zeta potential was less negative than that of the cores, but comparable to that of the FLS membrane‐derived vesicles (Figure 5B).

To verify the ability of FIRN to bind with activated T cells, fluorescently labeled FIRN were added to activated T cells. After incubation and washing, significant fluorescence was observed on T cells incubated with FIRN (Figure 5C). The protein expression of PD‐L1, Gal‐9, and FasL on cell membrane coated nanoparticles was also detected by western blotting (Figure 5D,E). Significantly increased expression of PD‐L1 and Gal‐9 was detected in FIRN compared with unstimulated FLS cell membrane coated nanoparticles (FLS‐MVs‐NPs). Furthermore, FasL expression was also detected on FIRN, suggesting multiple inhibitory pathways of FIRN on activated T cells.

Next, we investigated the effect of FIRN on limiting the activated T cells response. FIRN showed a remarkable ability in inhibiting activated T cell proliferation, and cytokine production, better than FLS‐MVs‐NPs and IFN‐*γ*‐FLS‐MVs‐NPs (Figure 5F–H). As IFN‐*γ* can also induce IL‐6 production in FLS, which is important for inducing Th17 differentiation, we also investigated the effect of FIRN on Th17 differentiation. In addition to the inhibition of proliferation, FIRN significantly decreased the production of IL‐17A by activated T cells (Figure 5H).

### 2.6 FIRN Can Target Inflammatory Joints and Alleviate Arthritis in CIA

**Figure 6:**
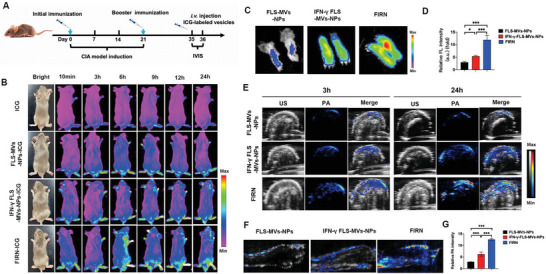
FIRN can target inflammatory joints in CIA mice. A) Illustration of the study of inflammatory joints targeting for ICG, ICG labeled nanoparticles from FLS‐MVs‐NPs, IFN‐*γ*‐FLS‐MVs‐NPs, or FIRN. B) In vivo imaging results of ICG, ICG labeled nanoparticles from FLS‐MVs‐NPs, IFN‐*γ*‐FLS‐MVs‐NPs, or FIRN after intravenous injection at the indicated times. C) Fluorescence change of FIRN, FLS‐MVs‐NPs, and IFN‐*γ*‐FLS‐MVs‐NPs penetrating into paws at 24 h post injection. D) The quantified analysis of relative fluorescence intensity (FL) intensity (*n* = 3 of each group). E) Penetration of different nanoparticles into the inflamed paws under ultrasound (US) and photoacoustics (PA) 3 h and 24 h after intravenous injection. F) Reconstructed 3D images of PA showed that FIRN could penetrate deeper into the inflamed tissues than that of FLS‐MVs‐NPs and IFN‐*γ*‐FLS‐MVs‐NPs at 24 h post injection. G) The quantified analysis of PA intensity (*n* = 3 of each group). *P*‐values were calculated using two‐sided unpaired *t*‐test (* *P* < 0.05, *** *P* < 0.001). MVs‐NPs: Cell membrane vesicles coated nanoparticles; FIRN: IFN‐*γ*‐rapamycin stimulated FLS derived cell membrane vesicles coated nanoparticles.

To investigate the ability of FIRN to target and concentrate in inflammatory joints in vivo, we established an animal model of RA by CIA,^[^ ^16^ ^]^ and indocyanine green (ICG) labeled FIRN was intravenously injected (**Figure** **6**A). In vivo fluorescence imaging experiments were performed at the indicated time points. Mice in the FIRN group exhibited the highest fluorescence signals in the inflamed paws, indicating excellent inflammatory site targeting of FIRN (Figure 6B). Fluorescence images of inflamed paws and the relative fluorescence intensity change further verified the FIRN targeting ability (Figure 6C,D) (Figure S4, Supporting information). To confirm the distribution of FIRN in the inflamed paws, we analyzed the photoacoustic (PA) signals and acquired ultrasound (US) images. PA signals of FIRN were visualized in the inflamed paws 3 h after injection; the signal was still observed at 24 h after injection (Figure 6E). 3D and quantitative analysis of the PA signal at 24 h after injection in the inflamed paws also confirmed that FIRN could target inflammatory sites in CIA (Figure 6F,G).

**Figure 7:**
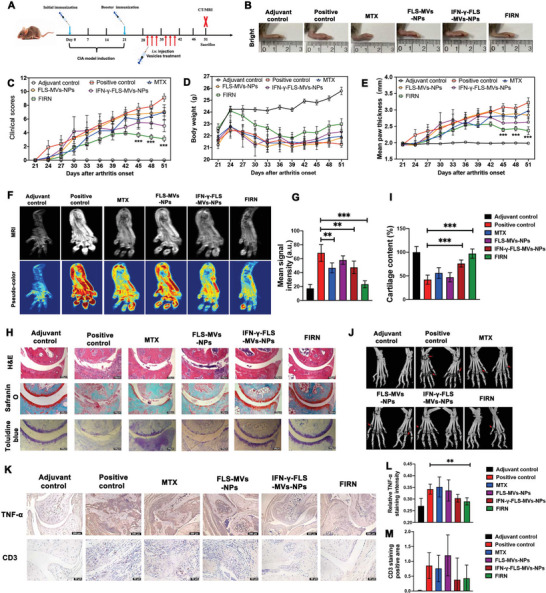
FIRN ameliorate inflammation and joint destruction in CIA mice. A) The study protocol of a therapeutic regimen with different treatments in CIA mice. Methotrexate (MTX, 20 mg kg^−1^ body weight; *n* = 7), FLS‐MV‐NPs (20 µg; *n* = 9), IFN‐*γ*‐FLS‐MVs‐NPs (20 µg; *n* = 5), or FIRN (20 µg; *n* = 8) were used to treat CIA mice every 2 days from 30 days after initial immunization. B) Representative image of inflamed paw in different groups on day 51 after the therapy. C) The clinical scores, D) the body weight change, and E) the mean paw thickness were detected after arthritis onset with different treatments. F) Magnetic resonance imaging (MRI) images of arthritic paws after therapies on day 51. G) Mean signal intensity of arthritic paws were calculated in different group. H) Representative images of H&E staining, Safranin‐O, and toluidine blue staining (Scale bars, 100 µm). I) Cartilage content were calculated in CIA mice treated with different group. J) Computed tomography (CT) images of paws after different therapy. K) Relative expression of TNF‐*α* in arthritic paws (Scale bars, 250 µm) and CD3 positive cell in synovium tissue (Scale bars, 50 µm) were detected by immunochemistry. L) Relative TNF‐ *α* staining intensity and M) CD3 staining positive area were calculated. The data are presented as the mean ± s.e.m. *P*‐values were calculated using two‐sided unpaired *t*‐test (** *P* < 0.01, *** *P* < 0.001). MVs‐NPs: Cell membrane vesicles coated nanoparticles; FIRN: IFN‐*γ*‐rapamycin stimulated FLS derived cell membrane vesicles coated nanoparticles; Adjuvant control: Mice immunized with adjuvant alone were used as control; Positive control: CIA mice treated with same volume of PBS as untreated control.

To explore the efficacy of FIRN in arthritis amelioration, the effect of FIRN was evaluated using a CIA model. Following the induction of arthritis, CIA mice developed joint swelling. As multiple joints are involved in RA as well as CIA, treatment strategies by injection of therapies in a single joint cannot alleviate systemic inflammation. Therefore, FIRN, FLS‐MVs‐NPs, and IFN‐*γ*‐FLS‐MVs‐NPs were injected intravenously (**Figure** **7**A). Methotrexate (MTX) has been the first‐line treatment for RA in clinical practice for a long time.^[^ ^17^ ^]^ CIA mice injected with MTX were used as controls. FIRN significantly alleviated arthritis in CIA mice (Figure 7B–E). At the treatment endpoint, joint inflammation was assessed using magnetic resonance imaging (MRI). FIRN significantly decreased the edema signal in inflamed joints (Figure 7F,G), suggesting that FIRN can alleviate inflammation in CIA mice. In addition, arthritic joints of CIA mice were sectioned for histological analysis. Bone damage, synovial hyperplasia, inflammatory cell infiltration, and pannus formation were observed following hematoxylin and eosin (H&E) staining in the positive control group. FIRN treatment significantly reduced the inflammatory cells infiltration and synovial hyperplasia, and protected against bone damage (Figure 7H). Safranin O staining and Toluidine blue staining also showed that FIRN significantly reduced cartilage destruction in CIA, and the effect of FIRN in protecting bone damage was much better than that of MTX therapy (Figure 7H,I). Bone erosion was further detected using micro‐computed tomography (CT) analysis. The results showed that FIRN had protective effects against bone erosion (Figure 7J). TNF‐*α* is one of the most important inflammatory cytokines in RA pathogenesis.^[^ ^18^ ^]^ Immunochemistry showed that FIRN treatment significantly reduced the TNF‐*α* expression in inflamed joints (Figure 7K,L). Also, aggregation of CD3^+^ T cells in inflamed synovium was observed in CIA mice, and FIRN treatment group showed decreased CD3^+^ T cells infiltration, though the difference was not significant (Figure 7K,M).

To investigate the safety of FIRN, H&E staining of the main organs was performed to determine whether the delivery of nanoparticles may give rise to toxicity in mouse models. After sacrifice, the heart, liver, spleen, lungs, and kidneys were dissected and appeared normal in appearance, color, and size. Furthermore, histological examinations demonstrated no apparent pathological damage in the FIRN treatment group (Figure S5, Supporting Information).

## 3 Discussion

**Figure 8:**
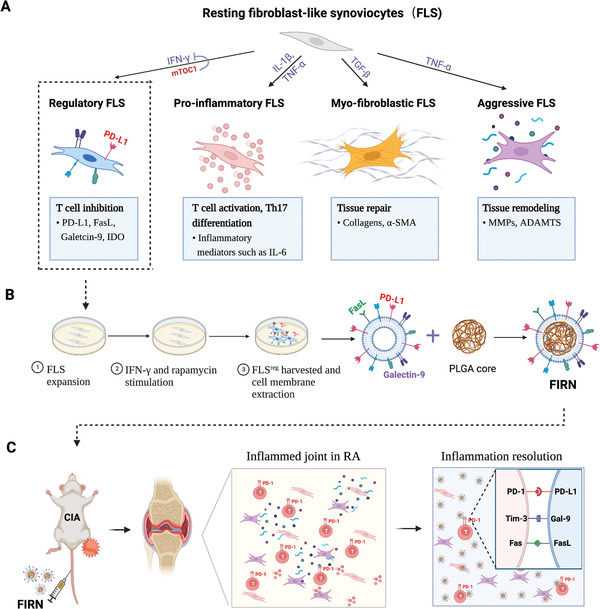
Schematic illustration depicting the study design. A) FLS subsets with distinct functional characteristics can be induced by different cytokines. B,C) Schematic illustration depicting the in vitro induction method for FLS^reg^, as well as the preparation and mechanisms of FIRN acting in RA treatment. Created with Biorender.com.

FLS play a central role in RA pathogenesis and are emerging as attractive therapeutic targets.^[^ ^9^, ^19^ ^]^ In the present study, we demonstrated for the first time that the “regulatory” phenotype of FLS (FLS^reg^) can be induced in vitro by using IFN‐*γ* plus rapamycin. The induced FLS^reg^ was characterized by high expression of PD‐L1 as well as several other inhibitory molecules that can interact with and limit the expanded PD1^+^ T cells in RA in a multi‐targeted manner. To avoid the loss of “regulatory” phenotype in vivo and separate dual effects of IFN‐*γ* on FLS, we proposed a novel targeted biomimetic treatment strategy for RA using FIRN. The shell of FIRN derived from the cell membrane of FLS stimulated with IFN‐*γ* and rapamycin, which naturally expressed several co‐inhibitory molecules, such as PD‐L1 and possible unknown inhibitory molecules, while avoiding the introduction of proinflammatory effect of IFN‐*γ* on FLS. In contrast to the high phenotypic plasticity of fibroblasts in vivo, this unique shell retained the regulatory characteristics of FLS induced in vitro and endowed FIRN with a stable capability to limit activated T cell responses at inflammatory sites in vivo (**Figure** **8**).

Over the years, cell membrane coated nanoparticles have been widely studied and applied as targeted drug delivery carriers in various diseases owing to their prolonged drug retention time and lower toxicity than nanoparticles.^[^ ^20^ ^]^ Concerning the application in RA, certain attempts have been made using cell membrane‐coated nanoparticles to treat RA in animal models. Zhang et al.^[^ ^21^ ^]^ used the neutrophils membrane‐encapsulated nanoparticles, which inherited the antigenic exterior and associated membrane functions of the source cells, to neutralize proinflammatory cytokines and suppress synovial inflammation, with good efficacy in CIA. Li et al.^[^ ^22^ ^]^ designed macrophage‐derived macrovesicle‐coated nanoparticles and showed that these particles could target inflammatory joints and alleviate arthritis in CIA mice. Our previous study found that tumor necrosis factor‐related apoptosis‐inducing ligand (TRAIL) expressing umbilical vein endothelial cell membrane‐based nanoplatforms can be applied for targeted anti‐arthritis therapy by binding and neutralizing multiple complex pathological factors vesicles.^[^ ^23^ ^]^ However, the pathogenesis of RA is complex, in which a variety of cells are involved, and the selection of the appropriate cell membrane for encapsulation to achieve high specificity and broad‐spectrum anti‐inflammatory effects still needs to be studied.

Fibroblast‐based therapy has been applied in clinical practice for dermal regeneration, such as the Food and Drug Administration (FDA) approved Dermagraft and LaViv, indicating its good accessibility in clinical applications. Fibroblasts are the primary cell type of connective tissue and may be utilized as a potentially more practical cell therapy than the widely applied mesenchymal stem cells, providing a new attractive therapeutic strategy for various diseases.^[^ ^24^ ^]^ In recent years, the potent immune regulatory ability of fibroblasts has been revealed. Fibroblasts are emerging as a promising therapeutic target in immune‐related diseases, including cancer, autoimmune disease, and infection.^[^ ^25^ ^]^ As the main cell component in the inflamed synovium of RA, FLS have a central role in RA pathogenesis, which intimately interact with T cells and mediate inflammation persistence. Using the FLS cell membrane as the shell of nanoparticles provides a unique targeting ability to inflamed joints due to its homotypic targeting ability.^[^ ^15^ ^]^

Besides its targeting ability, the IFN‐*γ* plus rapamycin induced FLS^reg^ cell membrane demonstrated a potent inhibitory effect on activated T cells both in vitro and in vivo, by multiple inhibitory pathways. RNA‐seq analysis of IFN‐*γ*‐activated fibroblasts revealed that the expression of various T cell inhibitory molecules was upregulated, including PD‐L1, Gal‐9, and FasL, whereas there was no obvious change in the stimulatory molecules. The PD‐1/PD‐L1 axis is recognized for its essential role in maintaining immune homeostasis by limiting activated T cells.^[^ ^6^ ^]^ Among the inhibitory molecules, we presently observed that PD‐L1 was most significantly upregulated, and blocking PD‐L1 significantly diminished the inhibitory effects of FLS on T cells. These findings indicate the important role of PD‐L1 in mediating the inhibitory effect of FLS^reg^ on T cells. However, blocking PD‐L1 cannot completely reverse the inhibitory effect of FLS^reg^ on T cells. This suggested other inhibitory pathways such as Gal‐9 and FasL on the induced FLS^reg^, as confirmed by western blotting, may also contribute to the inhibitory effect of FLS^reg^ on T cells.

Gal‐9 is a member of the galectin family of carbohydrate‐binding proteins and is an immune checkpoint molecule that plays an important role in sustaining T cells homeostasis.^[^ ^26^ ^]^ Functional studies have suggested that Gal‐9 can induce cell death in Th1 cells by binding to Tim‐3^[^ ^27^ ^]^ and promote the differentiation of Treg cells while suppressing Th17.^[^ ^28^ ^]^ Besides exerting effects via Tim‐3, Gal‐9 also controls CD40 induced proliferation of CD40^+^ T cells^[^ ^29^ ^]^ and regulates T cell death by interacting with PD‐1.^[^ ^30^ ^]^ FasL belongs to a large family of TNF‐like molecules. Binding of FasL to its Fas receptor activates the apoptosis signaling cascade.^[^ ^31^ ^]^ T cell receptor (TCR) stimulation increases Fas expression, making those activated T cells sensitive to apoptosis triggered by FasL, which is crucial for maintaining T cell homeostasis.^[^ ^32^ ^]^ Thus, the expression of Gal‐9 and FasL on the induced FLS^reg^ cell membrane may also contribute to the inhibitory effect on T cells.

The RNA‐seq analysis also showed that the expressions of TNF receptor superfamily member 14 (Tnfrsf14) and CD47 on fibroblasts were upregulated by IFN‐*γ*. Tnfrsf14, also known as herpes virus entry mediator, is a TNF‐receptor family member, which can bind to the co‐inhibitory receptor BTLA (B‐ and T‐ lymphocyte attenuator).^[^ ^33^ ^]^ In addition, Tnfrsf14 can bind to CD160, LIGHT, lymphotoxin‐alpha (LT*α*), and herpes simplex virus glycoprotein D. Tnfrs14 deliver both co‐stimulatory signal and co‐inhibitory signal when binding to different ligand.^[^ ^34^ ^]^ However, Tnfrsf14^−/−^ mice reportedly showed increased responses to T cell stimulation and enhanced susceptibility to auto‐immune disorders,^[^ ^35^ ^]^ suggesting that co‐inhibitory signaling overcomes its co‐stimulation counterpart. CD47 is a transmembrane protein known as integrin‐associated protein. Recent evidence has implicated CD47 as a novel innate checkpoint receptor target for cancer immunotherapy.^[^ ^36^ ^]^ CD47 plays a pivotal role in tissue homeostasis by delivering a “don't eat me” anti‐phagocytic signals upon binding to the signal‐regulatory protein alpha (SIRP*α*) receptor on myeloid cells, such as macrophages, to exert inhibitory effects directly and indirectly on T cells.^[^ ^37^ ^]^ The important role of Tnfrsf14 and CD47 in immune homeostasis suggests that the IFN‐*γ*‐induced FLS^reg^ may also inhibit T cells via these pathways; more importantly, the FLS^reg^ cell membrane may crosstalk with various other immune cells at the inflammatory site of RA in addition to T cells, which remains to be further studied.

IFN‐*γ* was originally identified as a macrophage‐activating factor that primes macrophages to differentiate into the classic inflammatory M1‐type macrophage.^[^ ^38^ ^]^ However, recent studies have shown that IFN‐*γ* has both proinflammatory and anti‐inflammatory effects.^[^ ^39^ ^]^ IFN‐*γ* is a pleiotropic cytokine with multiple effects that are important in tissue homeostasis.^[^ ^40^ ^]^ Results from the study on the impact of IFN‐*γ* on non‐leukocytes mainly linked IFN‐*γ* to pathways that result in the dampening of T cell responses. IFN‐*γ* secreted by metastases‐infiltrating lymphocytes leads to tumor evasion by upregulating negative immune checkpoints molecules, such as PD‐L1, which is an important mechanism in the immune escape of tumor cells.^[^ ^41^ ^]^ IFN‐*γ* also reportedly enhances the immunosuppressive function of MSCs^[^ ^42^ ^]^ and is a key factor in determining the efficacy of MSCs in RA treatment.^[^ ^43^ ^]^ In addition to PD‐L1, inhibitory molecules including Gal‐9,^[^ ^44^ ^]^ FasL,^[^ ^45^ ^]^ and CD47^[^ ^46^ ^]^ are reportedly upregulated by IFN‐*γ*. These reports indicate a critical role of IFN‐*γ* in the induction of immunosuppressive/regulatory functions in mesenchymal cells. Fibroblasts are the most abundant cells in mesenchymal tissues. The effect of IFN‐*γ* on inducing the regulatory phenotype of fibroblasts might be a conserved feedback mechanism in tissue immune homeostasis, which might be co‐opted in autoimmune diseases therapy.

In RA, both IFN‐*γ* and anti‐IFN‐*γ* have been used in RA treatment studies.^[^ ^47^ ^]^ It is still difficult to conclude the role of IFN‐*γ* in the pathogenesis of RA, which appears to have both proinflammatory and anti‐inflammatory effects. Several studies have demonstrated the anti‐inflammatory effects of IFN‐*γ* in RA. IFN‐*γ* can restrict the Th17 response and exacerbation of arthritis in an animal model of RA was found in IFN‐*γ* receptor‐ and IFN‐*γ*‐deficient mice.^[^ ^48^ ^]^ A defect in the IFN‐*γ* response has been found in lymphocytes of patients with RA.^[^ ^49^ ^]^ In the present study, IFN‐*γ* had dual effects on FLS; IFN‐*γ* can induce a regulatory phenotype by upregulating the expression of inhibitory molecules and also induce pro‐inflammatory characteristics of FLS by promoting IL‐6 production. The specific molecular mechanisms mediating the dual effects of IFN‐*γ* remain unclear. By using cell membranes derived from IFN‐*γ*‐activated FLS, we provide a method that may separate the dual effects of IFN‐*γ* and retain the anti‐inflammatory effect we need in autoimmune disease treatment.

Studies reported that IFN‐*γ* can inhibit activity or S6.^[^ ^50^ ^]^ In fibroblasts, we also showed IFN‐*γ* inhibited the phosphorylation of S6 kinase, and rapamycin enhanced the expression of PD‐L1 induced by IFN‐*γ*, indicating that rapamycin can reinforce the effect of IFN‐*γ*, potentially through inhibition of mTOC1. mTOR is an important signaling molecule that integrates diverse environmental inputs, including immune and metabolic signals, and subsequently directs cell growth and proliferation.^[^ ^51^ ^]^ mTOR signaling proceeds via mTORC1 (TOR Complex 1) and mTORC2. Rapamycin targets mTOR as part of an mTORC1. The activation of mTORC1 leads to the phosphorylation and activation of the ribosomal S6 kinase (S6K1).^[^ ^51^ ^]^ Rapamycin has long been used as an immunosuppressant in human transplant recipients, by inducing regulatory or tolerogenic phenotypes of immune cells, such as Tregs^[^ ^52^ ^]^ and tolerogenic dendritic cells.^[^ ^53^ ^]^ Effect of rapamycin on fibroblasts may be one of the new mechanisms involved in its immune tolerance induction effect. However, the specific molecular mechanisms underlying the role of mTOR in the regulatory fibroblast induction remain to be elucidated. In addition, future studies on the key pathways mediating the dual effects of IFN‐*γ* may help develop novel approaches to tease apart the proinflammatory effects from the anti‐inflammatory effects of IFN‐*γ* to design better therapeutics for autoimmune diseases.

## 4 Conclusion

FLS are the most important cells in the pathogenesis of RA and are specific to the joints. In this study, we demonstrated an in vitro induction method of FLS^reg^ using IFN‐*γ* and rapamycin. Based on the characteristics of FLS^reg^, we proposed an RA treatment strategy by using these cells. The cell membrane of FLS stimulated by IFN‐*γ* and rapamycin naturally expresses several co‐inhibitory molecules, such as PD‐L1, and possible as‐yet unknown inhibitory molecules. The application of the cell membrane as a shell can retain the anti‐inflammatory characteristics of FLS induced by IFN‐*γ* and rapamycin in vitro, while avoiding the introduction of pro‐inflammatory aspects of IFN‐*γ* on FLS. This novel approach effectively teased apart the anti‐inflammatory effect of IFN‐*γ* from its proinflammatory effect. We envision that the findings will facilitate the development of novel therapeutic strategies for autoimmune diseases based on the regulatory phenotype of fibroblasts.

## 5 Experimental Section

### Patients

This was reviewed and approved by the local ethical committee of the First Affiliated Hospital of Xiamen University (KY‐2019‐022). All patients and healthy volunteers provided written informed consents before participating. Blood and synovial fluid were collected from patients diagnosed with RA according to the 2010 Rheumatoid Arthritis Classification criteria.^[^ ^54^ ^]^ Twenty osteoarthritis (OA) patients and 11 healthy controls (HC) matched for age and sex were also recruited. The basic patient characteristics are shown in Table S1, Supporting Information. Mononuclear cells from the blood and synovial fluid were isolated, and the proportion of PD‐1 positive T cells was analyzed by FACS.

### RNA Isolation and Real‐Time PCR

Total RNA from stimulated cells was collected using TRIzol buffer (Invitrogen). Reverse transcription of RNA to cDNA was performed using a cDNA synthesis kit (Roche). Real‐time PCR was performed using FastStar University SYBR Green Master Mix (Roche) according to the manufacturer's instructions. The relative expression of mRNA was normalized to that of glycerladehyde 3‐phosphte dehydrogenase (GAPDH) using the 2^−∆∆Ct^ method. Primers used in this study are shown in **Table** **1**.

**Table 1:** Primers used in this study

Gene	Forward primer	Reverse primer
CD274	GGCAGGAGAGGAGGACCTTA	TTTGCGGTATGGGGCATTGA
Galctin‐9	CAGAGGTCAGAGTTCAAGGTGAT	CTTAGGGGTCCGTGGGAACT
FASL	TGAGTTCACCAACCAAAGCC	GAGTGGGGGTTCCCTGTTAAA
IDO	ATGAAGATGTGGGCTTTGCTCT	TATTGCGGGGCAGCACCTTT
CD80	CCTCGCTTCTCTTGGTTGGA	GGAGGGTCTTCTGGGGGTTT
CD86	CAGCACGGACTTGAACAACC	CTCCACGGAAACAGCATCTGA
ICOS‐L	GCTGCGTAGAGAATGTGGCT	TGAAGGAAACGAATGCCGCT
CD40	TTGTTGACAGCGGTCCATC	GGTGCAGTGTTGTCCTTCCT
GITR‐L	TACTTCACTCAAGCCAACTGC	CAGGAATCACTTGGCCGTAGA
CD70	TACAGCGCCTGACATACCTG	GGAGTTGTGGTCAAGGGCATA
OX‐40L	GCAAAGGACCCTCCAATCCA	TCGCACTTGATGACAACCGA
*α*SMA	CCTTCGTGACTACTGCCGAG	GCGTTCGTTTCCAATGGTGA
Collagen I	GTCTTGCTGGCCTACATGGT	AAAGTCATAGCCACCTCCGC
Collagen III	GAGGAATGGGTGGCTATCCG	TCGTCCAGGTCTTCCTGACT
Collagen IV	ATTAGCAGGTGTGCGGTTTG	CGATGAATGGGGCGCTTCTA
Collagen V	ATCCGAGGACAATCGGGTGA	GACCAACTGTGCCTGGATCA
IL‐6	GTCCTTCCTACCCCAATTTCCA	TAACGCACTAGGTTTGCCGA
TNF‐*α*	CCCACGTCGTAGCAAACCA	ACAAGGTACAACCCATCGGC
ICAM‐1	TGTCAGCCACCATGCCTTAG	CAGCTTGCACGACCCTTCTA
VCAM‐1	AATGACCTGTTCCAGCGAGG	TCACAGCCAATAGCAGCACA
GAPDH	AACTTTGGCATTGTGGAAGG	ACACATTGGGGGTAGGAACA
MMP3	CCCACATCACCTACAGGATTGT	GACTGTTCCAGGCCCATCAA
MMP9	CCTGGAACTCACACGACATCTTC	TGGAAACTCACACGCCAGAA
BAFF	TCCAGCAGTTTCACAGCGAT	GGTGTTGCTGAACCTCGGTA

### T Cell Activation and Co‐Culture with FLS

Naïve T cells from the spleen were isolated using a Pan T Cell Isolation Kit (Mitenyi Biotec) according to the manufacturer's instructions. T cells were labeled with 2.5 µm pre‐heated CFSE (Invitrogen) at 37 °C for 15 min and seeded at 2×10^5^ cells per well in 200 µL RPMI 1640 medium in a microtiter plate containing wells pre‐coated with anti‐CD3(1 µg mL^−1^) and anti‐CD28 (1 µg mL^−1^) antibodies (BioLegend) overnight. For the co‐culture experiment, FLS was added to T cells at a ratio of 1:10 (fibroblasts/T cells). To confirm the function of IFN‐*γ* or PD‐L1 in regulating the FLS phenotype, neutralizing anti‐IFN‐*γ* antibody (Ab) (10 µg mL^−1^) (Thermo Fisher Scientific), neutralizing anti‐PD‐L1 Ab (100 µg mL^−1^) (Abcam), and isotype control antibody (Thermo Fisher Scientific) were added to the co‐culture media.

### Flow Cytometry

T cell proliferation was detected by the dilution of CFSE on a flow cytometer. The percentage of cells with diluted CFSE was determined and expressed as the proliferation of T cells. For cell surface markers staining, the cells were resuspended in 200 µL PBS containing anti‐CD16/CD32 (BD Biosciences) and incubated for 15 min at room temperature. 2 µL of primary antibodies (anti‐CD274: phycoerthyrin [PE], anti‐CD3: Pecy7, anti‐CD80:PE; anti‐MHC‐II: fluorescein isothiocyantae [FITC], anti‐CD86: Pecy5.5; all from BioLegend and diluted 1:200 in PBS) were added and incubated at room temperature for another 20 min. Cells were washed twice with PBS, resuspended in 400 µL PBS, and analyzed using flow cytometry (Beckman Coulter). All the results were analyzed and presented using FlowJo software (BD Bioscience).

### Cytokine Determination

For analysis of cytokine production from T cells with or without FLS in vitro, co‐culture with FLS was performed as described above. Supernatants were harvested at 4 days of co‐culture. The levels of IFN‐*γ*, IL‐2, IL‐4, IL‐17A, and IL‐10 were measured using commercially available ELISA kits, according to the manufacturer's instructions (R&D Systems). The absorbance at 450 nm was measured using a microplate reader.

### Western Blotting

Immunoblotting was performed using whole‐cell lysates prepared in RIPA buffer containing a protease inhibitor and phosphatase inhibitor cocktail (Roche) on ice for 30 min. Protein was quantified using a bicinchoninic acid protein assay kit (Thermo Fisher Scientific). Thirty micrograms protein per lane was loaded and resolved by SDS‐PAGE. The proteins were transferred to polyvinylidene difluoride membranes (PVDF, Bio‐Rad). Membranes were incubated with primary antibodies overnight after blocking with 5% skim milk for 1 h. Membranes were then washed and incubated with horseradish peroxidase‐conjugated antibodies to the primary antibodies. The proteins were visualized using an ECL system (Bio‐Rad). Antibodies specific for the following proteins were purchased from Cell Signaling Technologies: STAT1, phosphorylated STAT1, ERK, phosphorylated ERK, p70S6, phosphorylated p70S6, Akt, phosphorylated Akt and β‐actin. Antibodies specific for PD‐L1, Galctin‐9, and FasL used for western blotting were purchased from Abcam.

### RNA‐Seq Analysis

Fibroblasts and IFN‐ *γ* stimulated fibroblasts were harvested using TRIzol reagent for RNA‐seq analysis. After total RNA was extracted, eukaryotic mRNA was enriched using Oligo(dT) beads. Prokaryotic mRNA was enriched by removing rRNA using the Ribo‐Zero Magnetic Kit (Epicentre, Madison, WI, USA). Then the enriched mRNA was fragmented into short fragments using fragmentation buffer and reverse transcribed into cDNA with random primers. Second‐strand cDNA was synthesized using DNA polymerase I, RNase H, dNTP, and buffer. Then the cDNA fragments were purified using the QiaQuick PCR extraction kit (QIAGEN, Venlo, The Netherlands), end‐repaired, poly(A) added, and ligated to Illumina sequencing adapters. The ligation products were size selected by agarose gel electrophoresis, PCR‐amplified, and sequenced using an Illumina HiSeq 2500 by Gene Denovo Biotechnology Co. The edgeR package (version 3.12.1) was used to identify differentially expressed genes across samples or groups. Genes with a fold change ≥2 and a false discovery rate <0.05 were identified in a comparison as significant DEGs. DEGs were then subjected to enrichment analysis by Gene Ontology and Kyoto Encyclopedia of Genes and Genomes databases.

### Derivation of Membranes

The murine synovial fibroblasts cell (FLS) line Mice‐synovial‐fibroblast (MSF) was purchased from the Qingqi (Shanghai) Biotechnology Development Co. Ltd. MSF cells were maintained in high‐glucose Dulbecco's modified Eagle medium supplemented with 10% heat inactivated FBS (Gibco). Rapamycin (10 µm, Sigma Aldrich) and/or 100 ng mL^−1^ IFN‐*γ* (Pepro Tech) were used to stimulate MSF cells for 24 h. Cells membrane nanoparticles preparation was performed as previously described.^[^ ^23^ ^]^ Cells were collected and lysed in Tris‐HCL buffer (pH = 7.4) containing 10 mm MgCl2, 1× phenylmethylsulphonyl fluoride (PMSF), 0.2 mm EDTA (all from MCE) and phosphatase inhibitor cocktail (Roche) on a shaker at 4 °C overnight. Cell lysate was sonicated three times with an ultrasonic probe at 20% amplitude for 20 s and then centrifuged three times at 500 g for 10 min at 4 °C. The supernatant solution was centrifuged at 10 000 × *g* for 30 min and further centrifuged at 70 000 × *g* for 90 min at 4 °C. The sediment was the cell membrane that suspended in PBS.

### Synthesis and Characterization of Nanoparticles

Cell membranes derived from FLS coated nanoparticles were prepared according to the previously published methods.^[^ ^23^ ^]^ Cell membranes were mixed with poly (DL‐lactic*‐co‐*glycolic acid) (50:50 PLGA, 0.67dlg‐1 lactel absorbable polymers) cores at the ration of 3:1 (w/w), followed by successively extrusion through 400 and 200 nm Nucleopore membranes (Whatman) using a mini‐extruder (Avanti polar lipids). The morphology of the cell membrane coated nanoparticles was observed using transmission electron microscope (TEM, JEM‐1200, Jeol Ltd, Japan) with uranyl acetate (0.2 wt%). FLS‐NPs were measured for hydrodynamic size and surface zeta potential with DLS (Malvern, UK). Surface markers of the FLS‐NPs were also detected by western blotting.

### T Cell‐NP Adhesion Assay

Naïve T cells and activated T cells which stimulated with anti‐CD3 and anti‐CD28 antibodies for 72 h were seeded in 24‐well plates. T cells were then incubated with MVs‐NPs, IFN‐*γ*‐MVs‐NPs, or FIRN labeled with Dil. After 1 h, 3 h, and 6 h of incubation, cells were collected and washed three times with cold PBS to remove free Dil. Cells were then stained with 4',6‐diamidino‐2‐phenylindole (DAPI) for 5 min. The cell sediment was suspended in 500 µL agarose gel (1%, W/V) and subsequently seeded in confocal dish (NEST). Images were obtained by laser scanning confocal microscope (Olympus FluoView FV 1000, USA).

### CIA

Male DBA/1J mice 6–8 weeks of age were purchased from Shanghai Slac Laboratory Animal Co. Ltd. The mice were raised in Xiamen University Laboratory Animal Center under pathogen‐free conditions. All procedures involving mice were reviewed and approved by the Committee of Xiamen University (XMULAC20190109). Mice were intradermally injected with 100 µg bovine type II collagen emulsified in complete Freund's adjuvant (CFA, containing 4 mg mL^−1^ of heat‐killed mycobacterium, Chondrex). Mice immunized with the adjuvant alone were used as adjuvant controls. A booster injection (100 µg of bovine type II collagen with incomplete Freund's adjuvant) was administered on day 21. Clinical scores were assessed every other day after disease onset. Clinical severity was graded as 0 (normal), 0.5 (erythema and edema in only one digit), 1 (erythema and mild edema of the footpad, or ankle or two to five digits), 2 (erythema and moderate edema of two joints [footpad or ankle, two to five digits]), 3 (erythema and severe edema of the entire paw), or 4 (reduced swelling and deformation leading to incapacitated limb). CIA mice with a score of 2 to 3 were randomly divided into three treatments groups: Free MTX (20 mg kg^−1^ body weight), FLS‐MVs‐NPs, IFN‐*γ*‐FLS‐MVs‐NPS and FIRN (20 µg per mice per 100 µL). They each received an intravenous injection. Saline injection was used as a positive control. The treatment was started on day 30 after the first immunization every 2 days for approximately six times.

### In Vivo Targeting Assays

For the fluorescence imaging (IVIS), the established CIA mice with an average score of 3 to 4 were divided randomly into four groups (*n* = 3 per group): free ICG, ICG labeled FLS‐MVs‐NPs, ICG labeled IFN‐*γ*‐MVs‐NPs and ICG labeled FIRN. Each treatment compound was suspended in 100 µL PBS containing 20 µg ICG and injected intravenously. The fluorescence images were obtained by IVIS system at the indicated time after injection. For the photoacoustic imaging (PAI), the mice also received the same injection as the IVIS assay. After the injection, the PAI images were acquired using the Vevo Laser platform. The relative fluorescence intensity was quantified using Living Image software.

### CT/MRI

Computer tomography (CT) images were used to evaluate the state of bone and joint damage with different therapeutics using an Inveon CT device (Siemens). The obtained data were processed to form the 3D structure of the joint. Magnetic resonance imaging (MRI) was used to evaluate the levels of inflammation using a 9.4 T Biospec scanner (Bruker).

### Histological Analysis of Knee Joint

Hind paws were collected after the mice were sacrificed and fixed overnight in 4% paraformaldehyde. The hind limbs were decalcified with 10% EDTA solution for 40 days and embedded in paraffin and sliced into 4 µm‐thick sections. Tissue sections were stained with H&E, Safranin‐O, and Toluidine blue. Immunohistochemistry was performed to detect immunoreactivity for TNF‐*α* and CD3 in sections using a rabbit anti‐mouse TNF‐ *α* antibody or rabbit anti‐mouse CD3 antibody (2.5 µg mL^−1^, Boster Biological Technology). 3,3′ Diaminobenzidine (DAB) (Beijing Zhongshan Golden Bridge Biological Technology) was used to detect the positive signals. Images of the sections were obtained using an IX71 light microscope (Olympus). The histological changes with synovial inflammation and cartilage erosion of the ankle joints were evaluated by HSS (histopathological scores of synovia). The relative TNF‐*α* staining intensity in tissue sections and CD3 positive area in synovial tissue were calculated with Image J Software (National Institutes of Health).

### Statistical Analysis

The results are expressed as the mean± SD of data obtained from at least three independent experiments in each experiment. Unpaired *t*‐tests and Spearman correlation analyses were performed using Prism 6.0 (GraphPad Software). All *P*‐values were two‐tailed. *P* < 0.05 was considered statistically significant (*P < 0.05, **P < 0.01, and ***P < 0.001).

## Conflict of Interest

The authors declare no conflict of interest.

## Supporting information

Supporting Information

## Data Availability

The data that support the findings of this study are available from the corresponding author upon reasonable request.
