# Supplementary material for: Regulatory Fibroblast‐Like Synoviocytes Cell Membrane Coated Nanoparticles: A Novel Targeted Therapy for Rheumatoid Arthritis
Source: Adv Sci (Weinh). 2022 Dec 12;10(4):2204998. doi: 10.1002/advs.202204998 (PMC9896074; doi:10.1002/advs.202204998)
Supplement: Supplementary file 1 — Supporting Information [file ADVS-10-2204998-s001.pdf]

ADVANCED SCIENCE
Open Access

Supporting Information

for *Adv. Sci.*, DOI 10.1002/advs.202204998

Regulatory Fibroblast-Like Synoviocytes Cell Membrane Coated Nanoparticles: A Novel Targeted Therapy for Rheumatoid Arthritis

Yuan Liu, Peishi Rao, Hongyan Qian, Yesi Shi, Shiju Chen, Jingying Lan, Dan Mu, Rongjuan Chen, Xinwei Zhang, Chaoqiong Deng, Gang Liu and Guixiu Shi**

Supporting Information

Title

Regulatory fibroblast-like synoviocytes cell membrane coated nanoparticles, a novel targeted therapy for rheumatoid arthritis

Yuan. Liu,[1, 2]*† Peishi. Rao,*[1, 2, 4]*† Hongyan. Qian,*[1, 2]*† Yesi. Shi,*[3]*† Shiju. Chen,*[1, 2] *Jingying. Lan,*[1, 2] *Dan. Mu,*[3] *Rongjuan. Chen,*[1, 2] *Xinwei. Zhang,*[1] *Chaoqiong. Deng,*[1, 2] *Gang. Liu,*[3*] *Guixiu. Shi*[1, 2*]

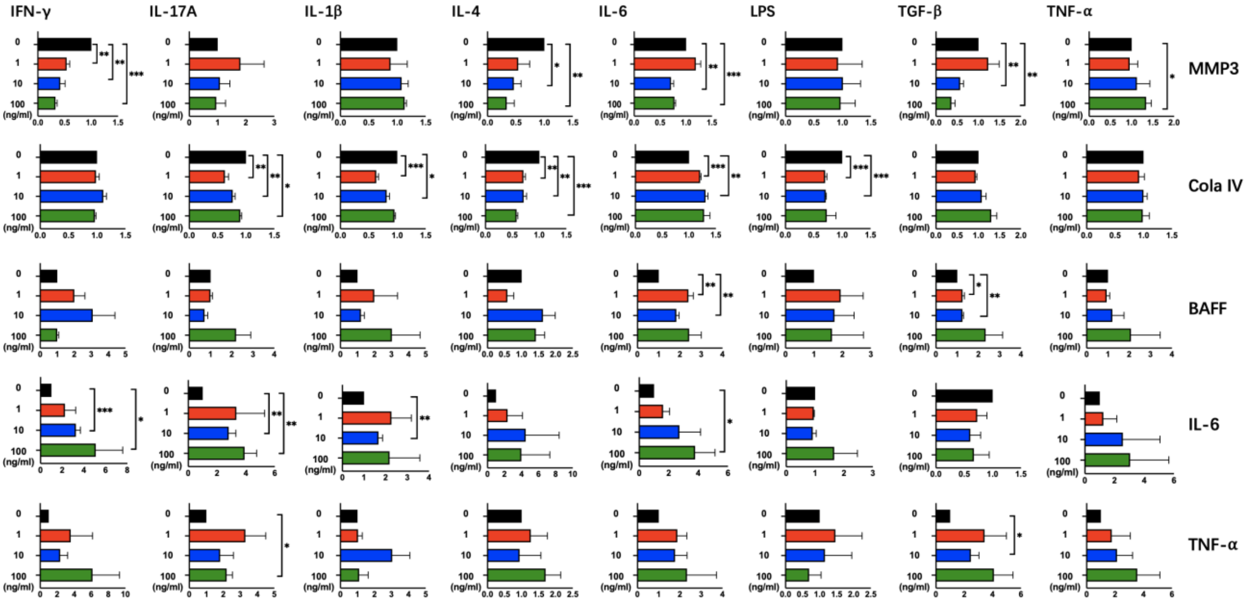

Figure. S1.

Phenotype of fibroblasts can be induced by different cytokines. FLS were treated with different concentration of TNF-α, IL-6, IL-1β, IFN-γ, IL-17A, IL-4, TGF-β, and LPS for 24 h. The relative expressions of MMP3, Cola-IV, BAFF, IL-6, and TNF-α in FLS were detected by RT- PCR. The data are presented as the mean ± sem (n= 3 independent experiments); * P<0.05, ** P<0.01, *** P<0.001.

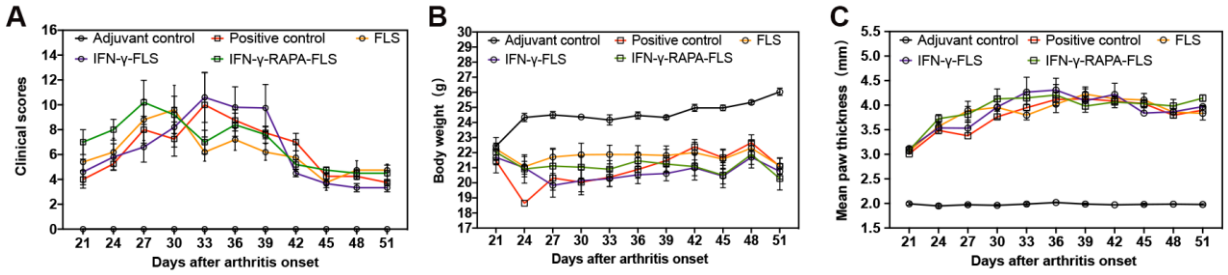

Figure. S2.

FLS^{reg} induced *in vitro* do not ameliorate inflammation in a CIA mouse model by direct application. CIA mice were induced and treated with FLS, IFN-γ-FLS, and IFN-γ-RAPA-FLS after arthritis onset. The clinical scores (**A**), the body weight change (**B**) and the mean paw thickness (**C**) were detected after arthritis onset with the different treatments.

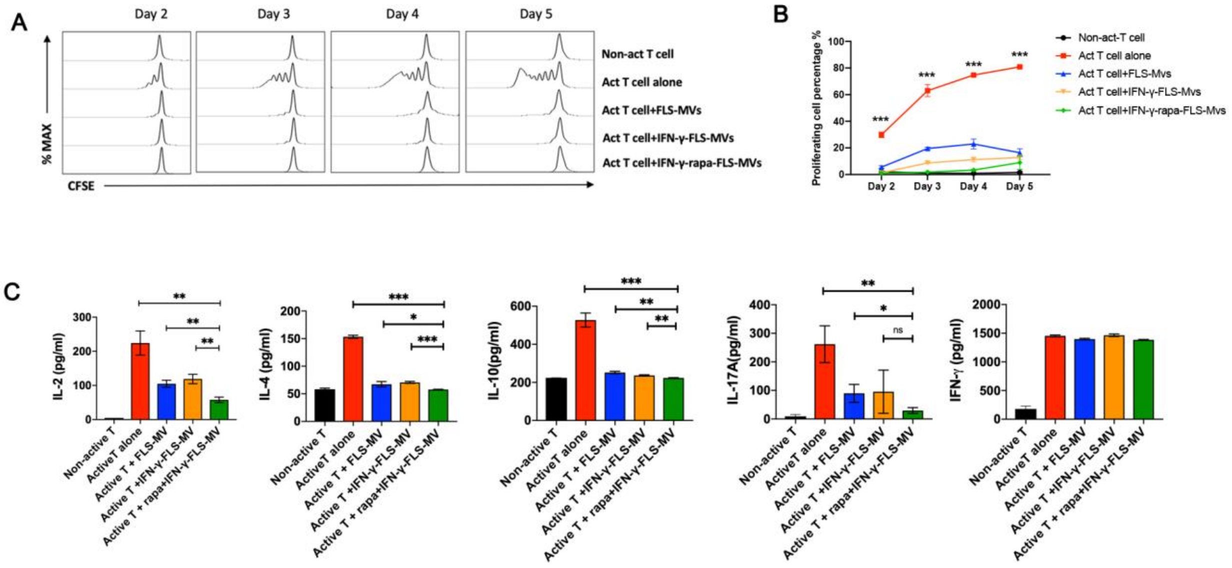

Figure. S3.

Cell membrane vesicles （MVs） derived from FLS under different stimulation inhibited the proliferation and activation of T cells *in vitro*. A and B. T cells activated with anti-CD3/CD28 (1 μg/ml) and incubated with MVs (50 μg/ml) derived from different treatment FLS. Proliferation rate of T cells were detected by FACS. **C.** Cytokines produced by T cells incubated with MVs were detected by ELISA. The data are presented as the mean ± s.e.m. (n= 3 independent experiments). P-values were calculated using two-sided unpaired t-test (* P<0.05, ** P<0.01, *** P<0.001).

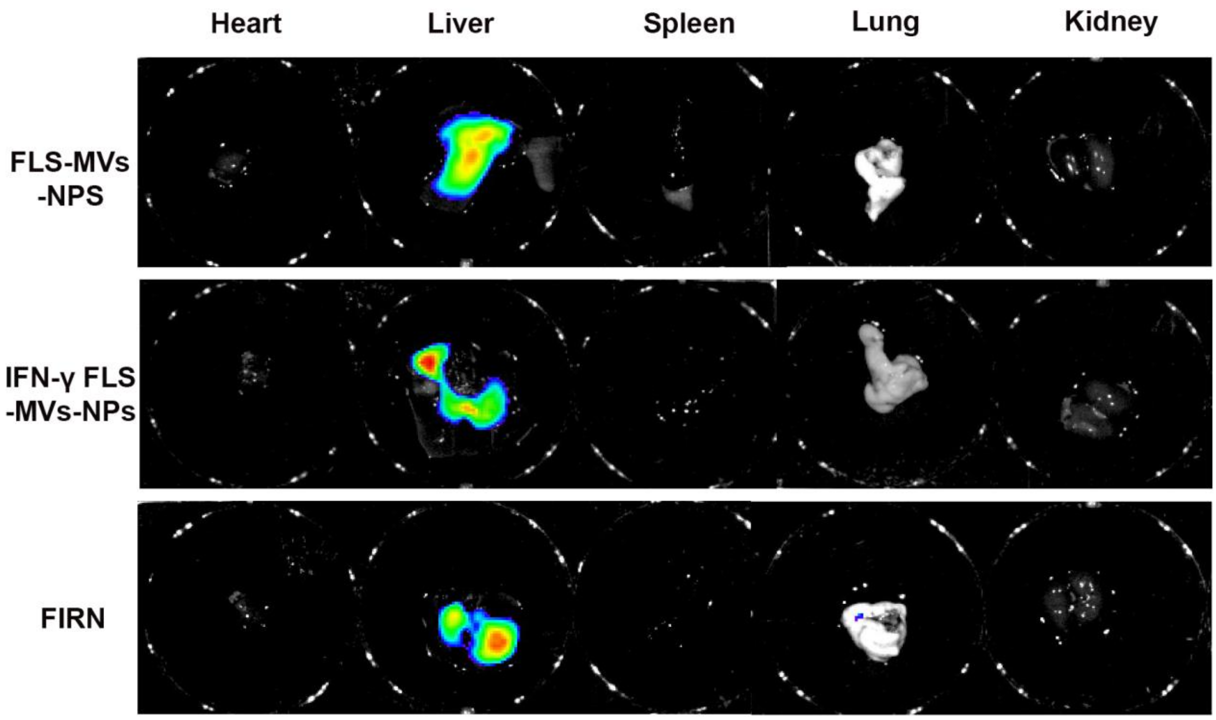

Figure. S4.

***In vivo* fluorescence images of nanoparticles penetrating in different organ.** The penetration of nanoparticles coated by FLS-MVs, IFN-γ-FLS-MVs or IFN-γ-Rapamycin-FLS-MVs into heart, liver, spleen, lung and kidney after 24h i.v. injection. MVs-NPs: Cell membrane vesicles coated nanoparticles. FIRN: IFN-γ-rapamycin stimulated FLS derived cell membrane vesicles coated nanoparticles.

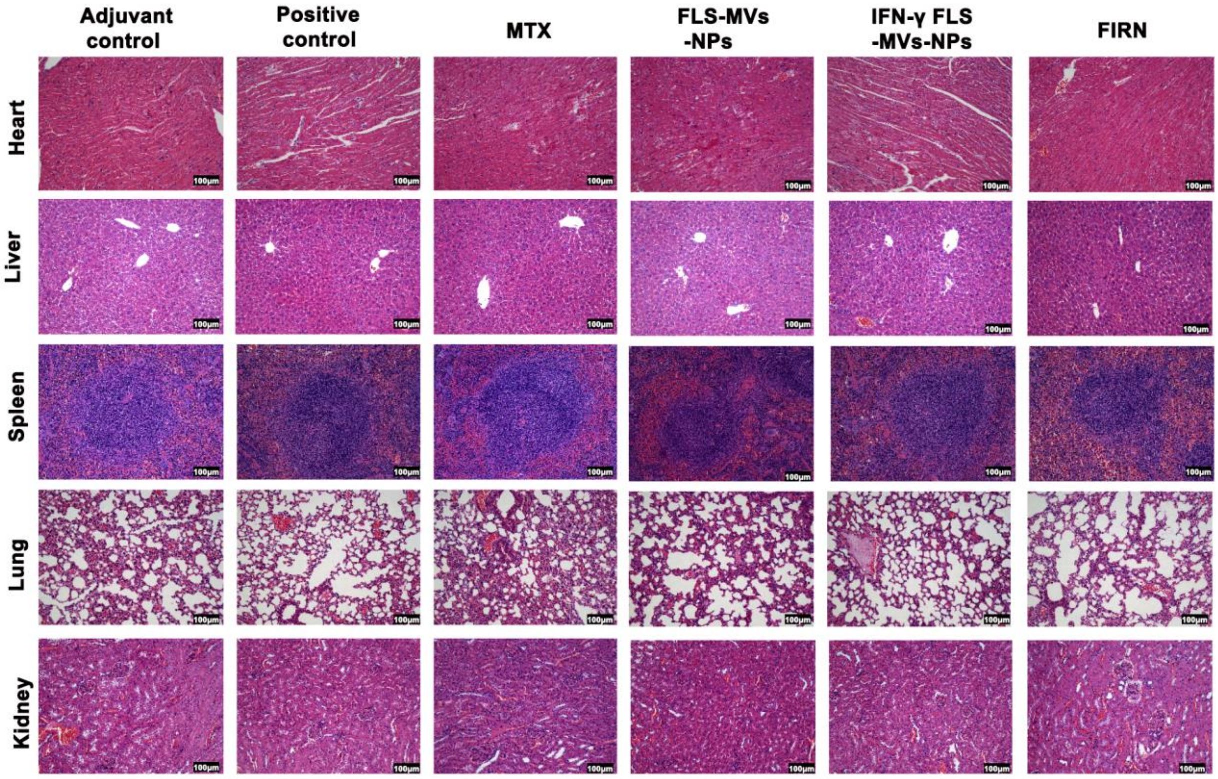

Figure. S5.

Histological analysis of organ damage in different treatment groups. H&E staining of heart, liver, spleen, lung and kidney was performed to evaluate the organ damage in different groups. (Scale bars, 100 μm). **MVs-NPs:** Cell membrane vesicles coated nanoparticles. **FIRN:** IFN-γ-rapamycin stimulated FLS derived cell membrane vesicles coated nanoparticles.

Table S1. Basic characteristics of patients in the present study.

	RA PBMC (n=40)	RA SF (n=9)	OA PBMC (n=20)	OA SF (n=7)
**Sex (female/male)**	33/7	8/1	14/6	5/2
**Age (years, mean ± SD)**	60.1±9.7	57.8±7.6	56.9±14.6	52.0±11.9
**Rheumatoid factor (positive/negative)**	30/6	7/2	2/16	0/1
**Anti-cyclic citrullinated peptide (positive/negative)**	24/3	7/0	4/11	0/0
